# One-step synthesis of *Lycopodium* alkaloid (-)-huperzine W via Suzuki-Miyaura coupling

**DOI:** 10.1007/s13659-012-0084-2

**Published:** 2012-12-14

**Authors:** Tao Xu, Shi-Zhi Jiang, Huai-Rong Luo, Yu-Rong Yang

**Affiliations:** 1 State Key Laboratory of Phytochemistry and Plant Resources in West China, Kunming Institute of Botany, Chinese Academy of Sciences, Kunming, 650201 China; 2 University of Chinese Academy of Sciences, Beijing, 100049 China

**Keywords:** total synthesis, huperzine W, lycopodium alkaloids

## Abstract

The first total synthesis of (−)-huperzine W (**1**) has been achieved. Key element of the synthesis is a highly convergent assemblage for the two rings system of target molecule utilizing an efficient Suzuki-Miyaura coupling reaction between chiral iodide **2** and 2-allylpyrrolidinone **4**. Evaluation of the AchE inhibition of synthetic huperzine W was also carried out.

**Electronic Supplementary Material:**

Supplementary material is available for this article at 10.1007/s13659-012-0084-2 and is accessible for authorized users.

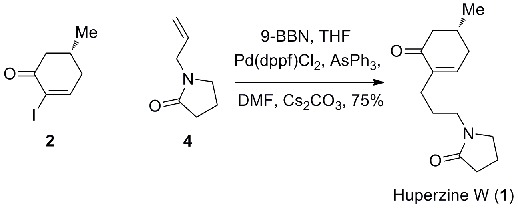

## Introduction

The *Lycopodium* alkaloids[1] are a diverse group of structurally complex natural products. Owing both to their appealing, synthetically challenging polycyclic systems with dense stereochemical array and wide-ranging biological activities, a wealth of total synthesis of *Lycopodium* alkaloids have been reported.[2] In the context of our ongoing synthetic endeavor on a complex polycyclic *Lycopodium* alkaloid,[3] huperzine W, an unusual structure-simplified natural *Lycopodium* alkaloid attracted our attention. Huperzine W, isolated in 2002 along with alopecuridine by Zhu[4] and co-workers from the plant of *Huperzia serrata*, is a novel 14 carbons *Lycopodium* alkaloid whose structure consists of a chiral cyclohexenone unit and a 2-pyrrolidinone moiety, linked by three carbon atoms. We were attracted to a synthesis of huperzine W not only because it has not yet to be synthesized, albeit architecturally rather simple, but also due to our interesting observation that the unique backbone of huperzine W is embedded delicately in the more intriguing polycyclic systems of other *Lycopodium* alkaloids (see Figure 1), such as 8-deoxyserratinine,[3b,3c,5] serratinine[6] and serratezomine A.[7] In principle, a step-economical and ideal synthesis of huperzine W, no doubt, will facilitate the total synthesis of the above complex alkaloids. Besides, we have a question about the biological activity of huperzine W, because its congener huperzine A is a well-known potent inhibitor of the enzyme acetylcholinesterase (AchE) used for the treatment of Alzheimer's disease.[8]

## Results and Discussion

Our retrosynthetic analysis of huperzine W (**1**) is outlined in

Scheme 1. We conjectured that an intermolecular Suzuki-Miyaura cross-coupling[9] of 2-allylpyrrolidinone **4** with iodide **2** would directly furnish the target molecule with just one single step. Both iodide **2** and 2-allylpyrrolidinone **4** are known compounds.[10] Iodide **2** was envisioned to arise from the optical cyclohexenone **3** and 2-allylpyrrolidinone **4** was prepared from pyrrolidinone **5** by N-alkylation.

Our synthesis commenced with the preparation of the two fragments. As shown in scheme 2, upon treatment with iodide and pyridine, the starting material enone **3**, prepared in multigram quantities according to the Caine's method,[11] smoothly yielded the $\alpha$-iodo enone **2**. On the other hand, 2-allylpyrrolidinone **4** can be prepared quantitatively through N-alkylation of pyrrolidinone **5** (NaH, allyl bromide).

*To whom correspondence should be addressed. E-mail: yangyurong@mail.kib.ac.cn

**Scheme 1.** Retrosynthetic analysis of huperzine W

Hydroboration of the 2-allylpyrrolidinone **4** with 9-BBN, then the *in situ* generated trialkylborane intermediate (not shown) was reacted with $\alpha$-iodo enone **2** under the standard Suzuki-Miyaura coupling conditions gave (–)-huperzine W (**1**) in 75% yield. All spectroscopic data matched those reported for the natural material. Our synthetic huperzine W (**1**) shows $[\alpha]_D^{25}$ –33 (*c* 1.28, $CHCl_3$), while the $[\alpha]_D$ value of the natural huperzine W is unavailable for comparison.

**Scheme 2.** Synthesis of huperzine W

Since the biological activity of huperzine W has not been documented in the isolation paper, we still wondered whether huperzine W might be active in inhibition of acetylcholinesterase (AChE), hoping to associate it with the potent huperzine A; and then evaluation of the AchE inhibition of synthetic huperzine W was performed. Unfortunately, this structural simplified *Lycopodium* alkaloid entirely lacks any activity in inhibition of AChE (see Table 1),[12] suggesting the unique chemical structure of huperzine A is not replaceable and essential for its pronounced bioactivity.

**Table 1. The AChE inhibitory activity of huperzine A, tacrine and huperzine W**

| Compound | Concentration ($\mu$M) | Inhibition Rate (%) |
|---|---|---|
| Huperzine A | 0.025 | 53 |
| Tacrine | 0.33 | 49 |
| **Huperzine W** | **100** | **2.3** |

In summary, the one-step and first asymmetric total synthesis of huperzine W was realized by a highly convergent route using an efficient Suzuki-Miyaura coupling reaction between chiral iodide **2** and 2-allylpyrrolidinone **4**. This step-economical synthesis is potentially beneficial to the synthesis of other relevant complex *Lycopodium* alkaloids.

## Experimental Section

**General Experimental Procedures.** All reactions were performed with dry solvents under anhydrous conditions, unless otherwise noted. Dry tetrahydrofuran (THF) were distilled over sodium-potassium alloy. Dichloromethane and dimethylformamide (DMF) were distilled over calcium hydride. Yields refer to chromatographically and spectroscopically homogeneous materials, unless otherwise noted. Reagents were used as received without further purification, unless otherwise stated. Silica gel (200–300 mesh, Qingdao Marine Chemical Ltd., China), light petroleum ether (bp 60–90 ºC), ethyl acetate and methanol were used for product purification by flash column chromatography. NMR spectra were recorded in $CDCl_3$ solution on Bruker AV-400 instrument with tetramethylsilane (TMS) as an internal reference. IR spectra were recorded with KBr pellets on a Bruker Tensor 27 FT-IR spectrometer. Optical rotations were determined on a Jasco P-1020 digital polarimeter. High-resolution mass spectral analysis (HRMS) data were recorded via electron impact mass spectrometry using a time of flight analyzer.

**Iodide (2).** To a solution of the optical cyclohexenone **3** (2.24 g, 20.3 mmol) in $CH_2Cl_2$ (200 mL) were added pyridine (20.7 mL, 257.8 mmol) and iodine (8.29 g, 32.5 mmol) at 0 °C. The mixture was allowed to raise to room temperature over 1 h. After 8 h, the mixture was diluted with $CH_2Cl_2$ and washed successively with 2N aqueous HCl, $H_2O$, saturated aqueous sodium hydrogen carbonate solution and saturated aqueous sodium thiosulfate solution. The organic phase was dried over sodium sulfate and concentrated. The crude product was purified by silica gel column chromatography (petroleum ether/ethyl acetate 50:1→40:1) to afford iodide **2** (4.08 g, 85% yield) as a white solid. $[\alpha]_D^{25}$ – 82 (*c* 4.04, $CHCl_3$); $^1$H NMR (400 MHz, $CDCl_3$) $\delta$ ppm: 7.69 (dd, *J* = 5.6, 2.8 Hz, 1H), 2.76–2.65 (m, 1H), 2.49–2.39 (m, 1H), 2.32–2.21 (m, 2H), 2.18–2.09 (m, 1H), 1.04 (d, *J* = 6.0 Hz, 3H); $^{13}$C NMR (100 MHz, $CDCl_3$) $\delta$ ppm: 192.5, 158.6, 103.4, 45.0, 37.9, 30.3, 20.6; IR (KBr) $\nu_{max}$ $cm^{-1}$: 2958, 2923, 1684, 1589, 879, 549; HRMSEI m/z: calcd for $C_7H_9IO$ $[M]^+$: 235.9698, found 235.9697.

**2-Allylpyrrolidinone (4).** To a suspension of sodium hydride (890 mg, 70% oil dispersion, 26.0 mmol) in THF (100 mL) was added slowly a solution of pyrrolidinone **5** (2.02 g, 23.6 mmol) in THF (100 mL) at 0 °C. The mixture was stirred for 1 h at 0 °C under argon atmosphere. Then allyl bromide (2.7 mL, 30.7 mmol) was added slowly at 0 °C. Then the mixture was stirred overnight at 75 °C. The reaction was quenched with saturated aqueous ammonium chloride solution and extracted with ethyl acetate. The combined organic extract was washed with brine, dried over sodium sulfate and concentrated. The crude product was purified by silica gel column chromatography (petroleum ether/ethyl acetate 2:1→1:1) to afford 2-allylpyrrolidinone **4** (3.53 g, 100% yield) as a colorless oil. $^1$H NMR (400 MHz, $CDCl_3$) $\delta$ ppm: 5.64–5.52 (m, 1H), 5.08–5.01 (m, 2H), 3.75 (d, *J* = 5.6 Hz, 2H), 3.23 (t, *J* = 7.2 Hz, 2H), 2.27 (t, *J* = 8.0 Hz, 2H), 1.94–1.84 (m, 2H); $^{13}$C NMR (100 MHz, $CDCl_3$) $\delta$ ppm: 174.6, 132.3, 117.6, 46.6, 45.0, 30.8, 17.6; IR (KBr) $\nu_{max}$ $cm^{-1}$: 2920, 1687, 1661, 1643, 1496, 1464, 1442, 1419, 1270, 1202, 996. HRMSEI m/z: calcd for $C_7H_{11}NO$ $[M]^+$:125.0841, found 125.0833.

**(–)-Huperzine W (1).** To a solution of 2-allylpyrrolidinone **4** (202 mg, 1.6 mmol) in THF (10 mL) was added a solution of 9-BBN (4.3 mL, 0.5 N, 2.1 mmol) at room temperature. After stirring for 4 h at room temperature, the mixture was treated with degassed water (0.44 mL) and transferred via cannula to a mixture of iodide **2** (251 mg, 1.1 mmol), Pd(dppf)$Cl_2$ (87 mg, 0.11 mmol), $AsPh_3$(33 mg, 0.11 mmol) and $Cs_2CO_3$ (658 mg, 2.0 mmol) in degassed DMF (3 mL). The mixture was stirred at room temperature for 1 h. Then the reaction was quenched with saturated aqueous ammonium chloride solution and extracted with ethyl acetate. The combined organic extract was washed with water, dried over sodium sulfate and concentrated. The crude product was purified by silica gel column chromatography (ethyl acetate/methanol 20:1) to afford (–)-Huperzine W **1** (187 mg, 75% yield) as a pale yellow oil. $[\alpha]^{25}_{D}$ – 33 (*c* 1.28, $CHCl_3$); $^1$H NMR (400 MHz, $CDCl_3$) $\delta$ ppm: 6.65 (br. d, *J* = 2.4 Hz, 1H), 3.34–3.27 (m, 2H), 3.20–3.13 (m, 2H), 2.42–2.24 (m, 4H), 2.15–2.01 (m, 3H), 2.02–1.87 (m, 4H), 1.58–1.47 (m, 2H), 0.95 (d, *J* = 6.0 Hz, 3H); $^{13}$C NMR (100 MHz, $CDCl_3$) $\delta$ ppm: 199.7, 175.0, 145.5, 138.3, 47.0, 46.6, 42.0, 34.3, 31.1, 30.6, 26.9, 26.2, 21.2, 17.9. IR (KBr) $\nu_{max}$ $cm^{-1}$: 2954, 2926, 1674, 1461, 1428, 1381, 1287, 1116, 1091, 1040, 922, 904. HRMSEI m/z: calcd for $C_{14}H_{21}NO_2$ $[M]^+$: 235.1572, found 235.1583.

**Inhibition of AChE Activity Assays.** The AChE inhibitory activity of synthetic (–)-Huperzine W **1** was assayed by the colorimetric method of Ellman.[13] Acetylthiocholine iodide (ATC), 5,5′-Dithiobis(2-nitrobenzoic acid) (DTNB), tacrine and AChE were purchased from Sigma. AchE (0.4 U/mL), ATC (6.25 mM) and DNTB (6.25 mM) were dissolved in 0.1M phosphate buffer (pH 8.0, respectively). Inhibitors (100 $\mu$M) were dissolved in dimethyl sulfoxide. The final dimethyl sulfoxide in all assays were maintained at 0.1% (v/v), including controls. Inhibitor solution (10 $\mu$L) and AChE (40 $\mu$L) were mixed in a test tube, and the tube was set on the incubator (30 °C). To the tube were added DTNB (20 $\mu$L), buffer (110 $\mu$L), and ATC (20 $\mu$L). The mixture was incubated at 30 °C for 30 min. The absorbance at 405 nm was measured spectrophotometrically. All test and control assays were corrected by blanks for nonenzymic hydrolysis. All experiments were done in triplicate with tacrine as a positive control.

## Electronic Supplementary Material

Supplementary material is available in the online version of this article at http://dx.doi.org/ 10.1007/s13659-012-0084-2 and is accessible for authorized users.

## Acknowledgments

We would like to thank the National Natural Science of China (No.20802084, 21072200), Programs of "One Hundred Talented People" and "Western Light" Joint Scholar of Chinese Academy of Sciences for financial support.

**Open Access** This article is distributed under the terms of the Creative Commons Attribution License which permits any use, distribution, and reproduction in any medium, provided the original author(s) and source are credited.

## Electronic supplementary material

Supplementary material, approximately 497 KB.
