# Supplementary material for: One-step synthesis of Lycopodium alkaloid (-)-huperzine W via Suzuki-Miyaura coupling
Source: Nat Prod Bioprospect. 2012 Dec 14;2(6):255–7. doi: 10.1007/s13659-012-0084-2 (PMC4131606; doi:10.1007/s13659-012-0084-2)
Supplement: Supplementary file 1 — Supplementary material, approximately 497 KB. [file 13659_2012_84_MOESM1_ESM.pdf]

Electronic Supplementary Material

One-step synthesis of Lycopodium alkaloid (–)-huperzine W via Suzuki-Miyaura coupling

Tao XU,[a,b] Shi-Zhi JIANG,[a] Huai-Rong LUO,[a] and Yu-Rong YANG[a,]*

[a]State Key Laboratory of Phytochemistry and Plant Resources in West China, Kunming Institute of Botany, Chinese Academy of Sciences, Kunming 650201, China

[b]University of Chinese Academy of Sciences, Beijing 100049, China

Received 19 October 2012; Accepted 9 November 2012

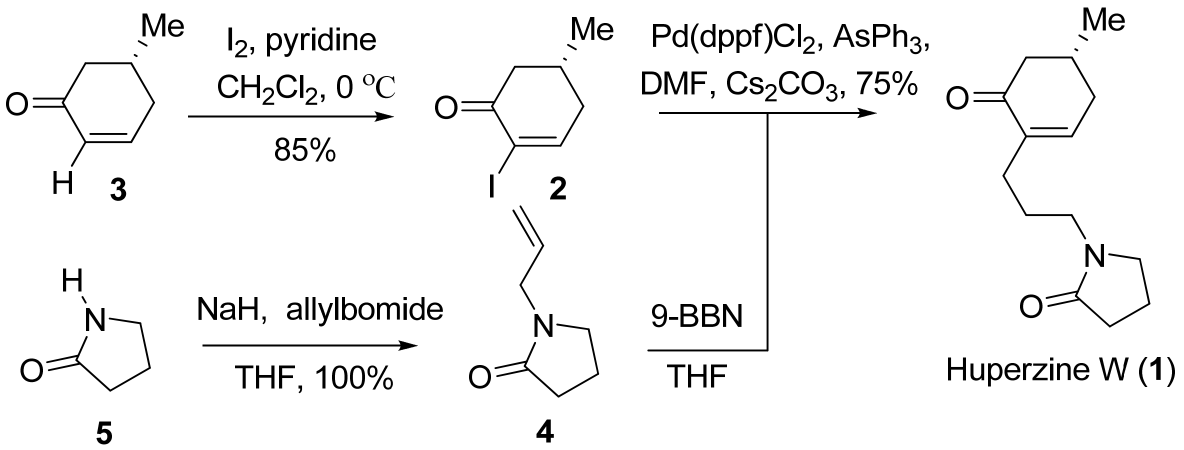

*To whom correspondence should be addressed. E-mail: yangyurong@mail.kib.ac.cn

Table of Contents

**I) Comparison of natural and synthetic (-)-Huperzine W**	**S3**
**II) NMR Spectra**	**S4-S6**

I) Comparison of Natural and Synthetic (-)-Huperzine W

δ_H (ppm)		δ_C (ppm)	
Natural (-)-Huperzine W	Synthetic (-)-Huperzine W	Natural (-)-Huperzine W	Synthetic (-)-Huperzine W
6.73 (dd, 5.6, 2.6, 1H)	6.65(br d, 2.4, 1H)	199.65	199.7
3.38 (t, 7.1, 2H)	3.34 – 3.27 (m, 2H)	174.99	175.0
3.25 (t, 7.2, 2H)	3.20 – 3.13 (m, 2H)	145.38	145.5
2.47 (ddd, 15.1, 3.0, 1.6, 1 H)	2.42 – 2.24 (m, 4H)	138.39	138.3
2.40 (br d, 14.9, 1H)	from above	46.99	47.0
2.37 (t, 8.1, 2H)	from above	46.65	46.6
2.16 (m, 1H)	2.15 – 2.01 (m, 3H)	42.04	42.0
2.15 (t, 7.6, 2H)	from above	34.36	34.3
2.08 (dd, 15.1, 11.5, 1H)	2.02 – 1.87 (m, 4H)	31.12	31.1
2.02 (overlapped, 1H)	from above	30.62	30.6
2.00 (q, 7.6, 2H)	from above	26.96	26.9
1.61 (q, 7.4, 2H)	1.58 – 1.47 (m, 2H)	26.22	26.2
1.03 (d, 6.3, 3H)	0.95 (d, 6.0, 3H)	21.13	21.2
		17.93	17.9

II) **NMR Spectra**

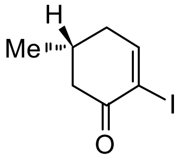

(2)

^{1}H-NMR ($CDCl_3$, 400 MHz)

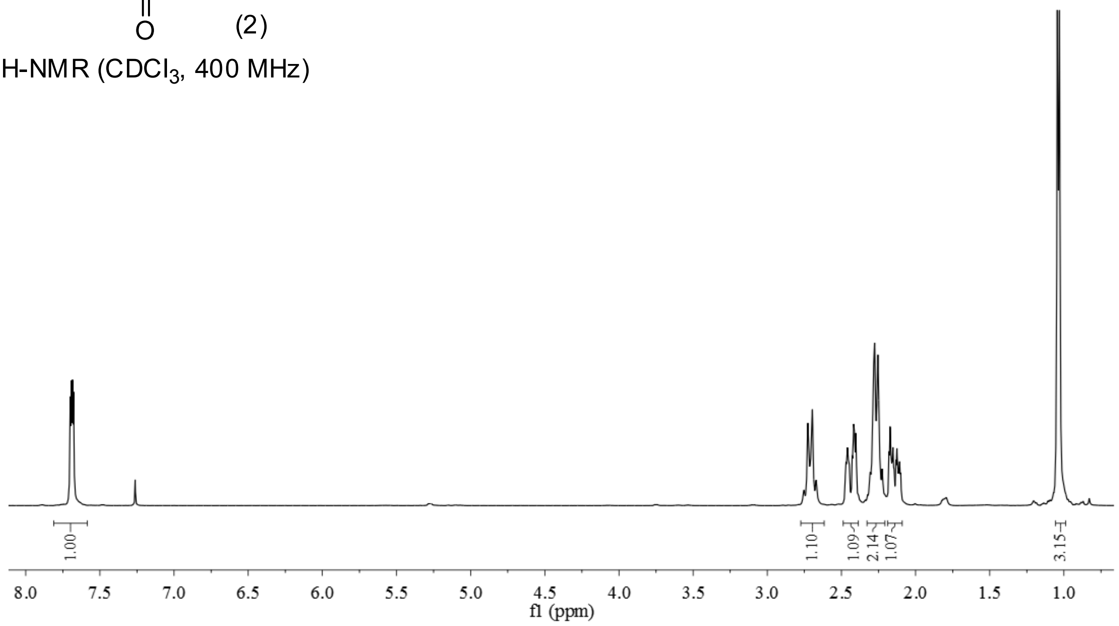

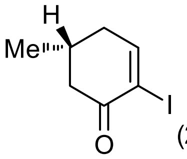

(2)

^{13}C-NMR ($CDCl_3$,100 MHz)

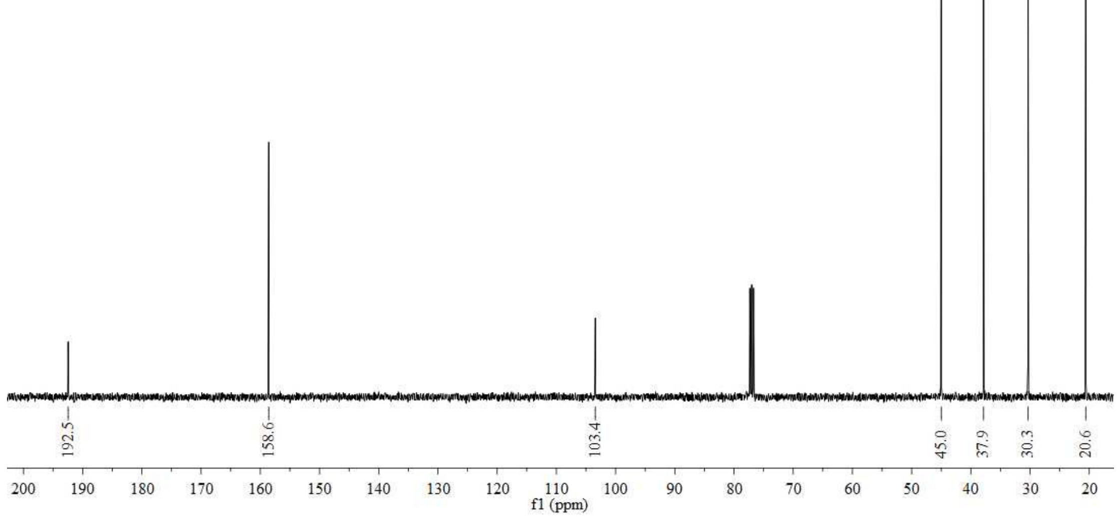

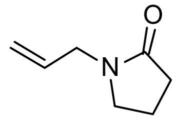

(4)

^{1}H-NMR ($CDCl_3$, 400 MHz)

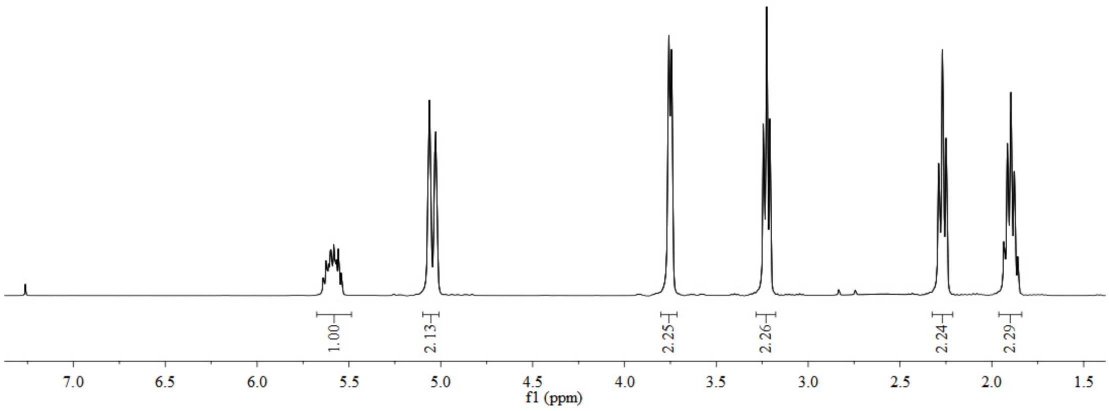

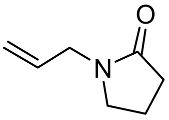

(4)

^{13}C-NMR ($CDCl_3$,100 MHz)

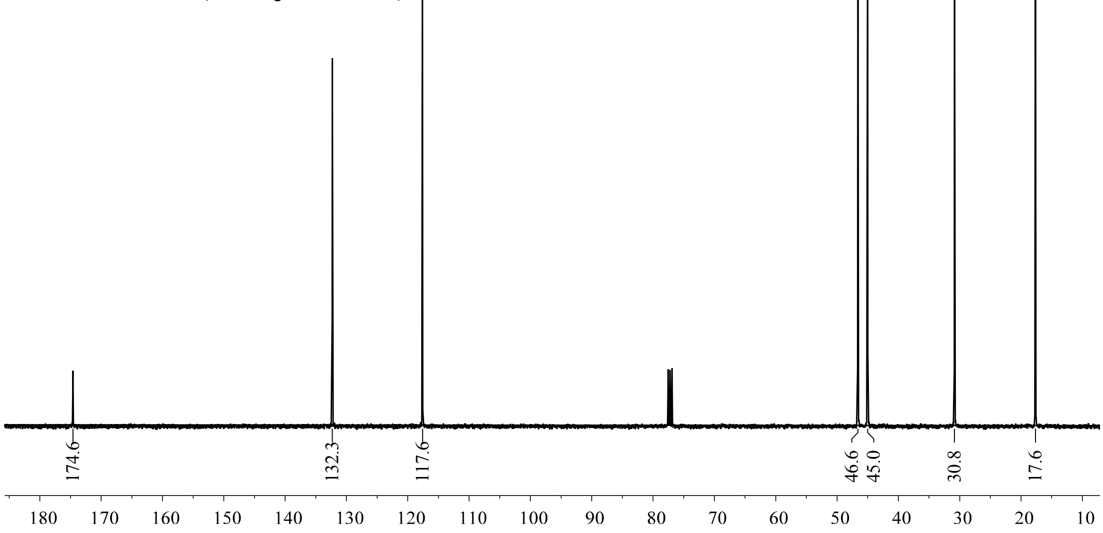

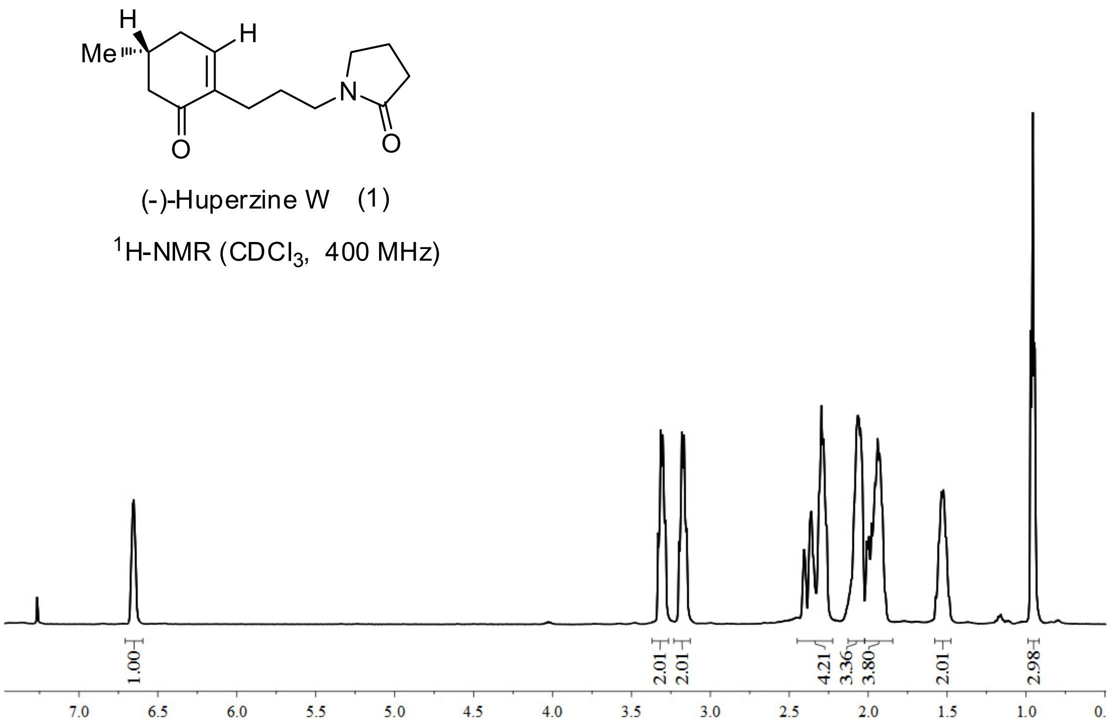
(-)-Huperzine W (1)
1H-NMR (CDCl3, 400 MHz)
1.00
2.01
2.01
4.21
3.36
3.80
2.01
2.98

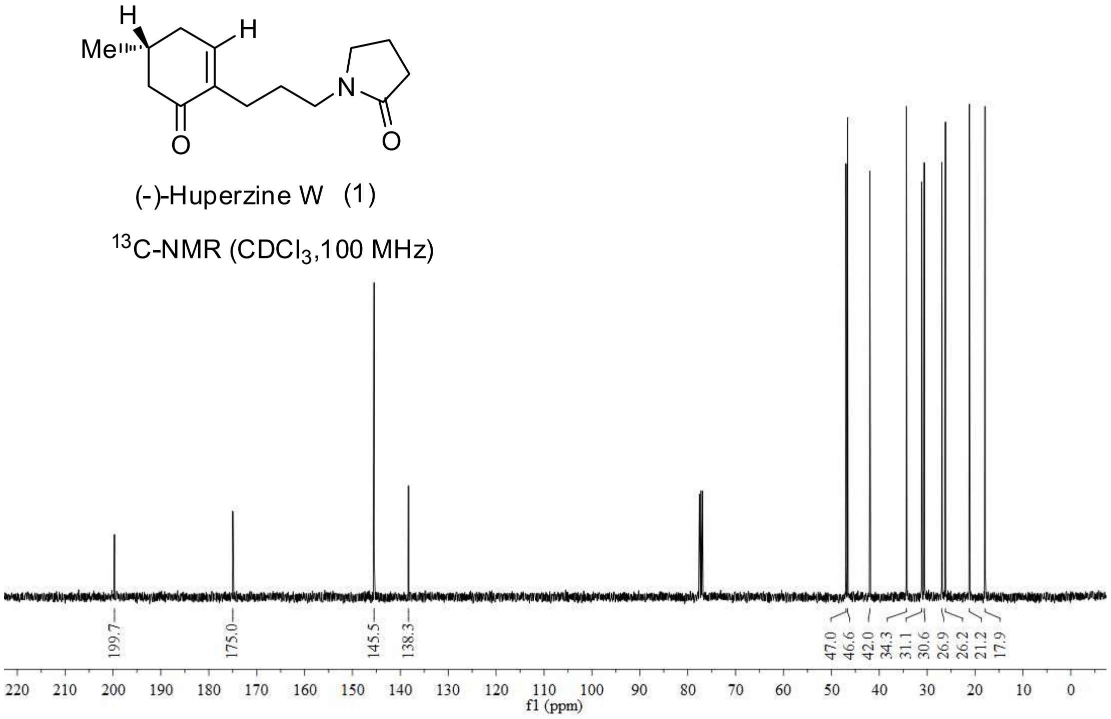
(-)-Huperzine W (1)
13C-NMR (CDCl3,100 MHz)
199.7
175.0
145.5
138.3
47.0
46.6
42.0
34.3
31.1
30.6
26.9
26.2
21.2
17.9
f1 (ppm)
